# Promoter methylation of *MGMT*, *MLH1* and *RASSF1A* tumor suppressor genes in head and neck squamous cell carcinoma: Pharmacological genome demethylation reduces proliferation of head and neck squamous carcinoma cells

**DOI:** 10.3892/or.2012.1624

**Published:** 2012-01-09

**Authors:** DIMITRIOS KOUTSIMPELAS, WARUT PONGSAPICH, ULF HEINRICH, SYLVIA MANN, WOLF J. MANN, JÜRGEN BRIEGER

**Affiliations:** Department of Otorhinolaryngology, Head and Neck Surgery, University Medical Center of the Johannes Gutenberg University Mainz, 55101 Mainz, Germany

**Keywords:** O-6-methylguanine-DNA methyltransferase, mutL homolog 1, Ras association domain family member 1, tumor suppressor gene, head and neck squamous cell carcinoma, 5-azacytidine

## Abstract

Promoter hypermethylation of tumor suppressor genes (TSGs) is a common feature of primary cancer cells. However, to date the somatic epigenetic events that occur in head and neck squamous cell carcinoma (HNSCC) tumorigenesis have not been well-defined. In the present study, we analyzed the promoter methylation status of the genes mutL homolog 1 (*MLH1*), Ras-association domain family member 1 (*RASSF1A*) and O-6-methylguanine-DNA methyltransferase (*MGMT*) in 23 HNSCC samples, three control tissues and one HNSCC cell line (UM-SCC 33) using methylation-specific PCR (MSP). The expression of the three proteins was quantified by semi-quantitative immunohistochemical analysis. The cell line was treated with the demethylating agent 5-azacytidine (5-Aza) and the methylation status after 5-Aza treatment was analyzed by MSP and DNA sequencing. Proliferation was determined by Alamar blue staining. We found that the *MGMT* promoter in 57% of the analyzed primary tumor samples and in the cell line was hypermethylated. The *MLH* promoter was found to be methylated in one out of 23 (4%) tumor samples while in the examined cell line the *MLH* promoter was unmethylated. The *RASSF1A* promoter showed methylation in 13% of the tumor samples and in the cell line. *MGMT* expression in the group of tumor samples with a hypermethylated promoter was statistically significantly lower compared to the group of tumors with no measured hypermethylation of the *MGMT* promoter. After treatment of the cell line with the demethylating agent 5-Aza no demethylation of the methylated *MGMT* and *RASSF1A* genes were determined by MSP. DNA sequencing verified the MSP results, however, increased numbers of unmethylated CpG islands in the promoter region of *MGMT* and *RASSF1A* were observed. Proliferation was significantly (p<0.05) reduced after treatment with 5-Aza. In summary, we have shown promoter hypermethylation of the tumor suppressor genes *MGMT* and *RASSF1A* in HNSCC, suggesting that this epigenetic inactivation of TSGs may play a role in the development of HNSCC. 5-Aza application resulted in partial demethylation of the *MGMT* and *RASSF1A* TSGs and reduced proliferation of the tumor cells suggesting further evaluation of 5-Aza for HNSCC treatment.

## Introduction

Head and neck squamous cell carcinoma (HNSCC) is a common neoplasm associated with exposure to tobacco and alcohol. It accounts for up to 5% of all newly diagnosed malignancies worldwide and is the sixth most common cancer in the world (1). The incidence of this type of tumor is expected to rise as a result of the increasing number of female and adolescent smokers. Despite considerable advances in diagnosis and treatment of HNSCC, the overall survival rate has remained constant at 60% over the past 30 years in the United States (2). The lack of progress in head and neck oncology emphasizes the need for basic studies on the molecular biology of HNSCC.

In HNSCC genetic analyses demonstrated the frequent loss of genomic material at several chromosomal loci suggesting the involvement of diverse tumor suppressor genes (TSGs) in the genesis of HNSCC (3,4). More recent, in addition to genetic alterations, promoter hypermethylation has been recognized as another mechanism of TSG inactivation. Accordingly, several studies have shown that methylation of CpG islands located within the promoter regions of tumor suppressor genes is a frequent event in the development of HNSCC and other human malignancies (5–7).

Among multiple TSGs that have been found to be hypermethylated in a variety of malignancies are the *RASSF1A* and the *MLH1* at chromosome arm 3p (8,9) and the *MGMT* TSG at 10q (10).

*RASSF1A* is a member of a new group of RAS effectors which are involved in cell cycle control, microtubule stabilization, cellular adhesion and motility as well as apoptosis. *RASSF1A* acts as a TSG by controlling mitotic function and decreasing the risk of aneuploidy leading to increased genomic stability (11). *MLH1* is a mismatch repair gene, functions to correct replicate mismatches that escape DNA polymerase proofreading, and hence plays an important role in the maintenance of genetic stability (12). The *MGMT* TSG is a DNA-repair gene, which prevents the alkylation of guanine (13).

To date, reports have revealed varying frequencies of TSG silencing by hypermethylation in HNSCC. The usefulness of the analysis of the promoter methylation status for prognostic purposes has been shown (14,15) as well as normalization of hypermethylation by drugs in cancer patients. e.g., the efficiency of 5-azacytidine (5-Aza, Vidaza^®^) and decitabine (Dacogen^®^) are established for the therapy of acute myeloid leukemia and myelodysplastic syndromes (16,17).

Considering these findings, we examined the methylation status and the expression of *MGMT*, *MLH1* and *RASSF1A* in 23 HNSCC biopsy samples and in one HNSCC cell line to establish a potential role of the hypermethylated TSGs in HNSCC development. Furthermore, we investigated the possibility of restoring the methylation status of the TSGs by treatment with 5-Aza and the functional impact of 5-Aza treatment on proliferation of the tumor cells.

## Materials and methods

### Patients and specimens

A total of 23 patients (19 males, 4 females) with histological confirmed squamous cell carcinoma and one HNSCC cell line were included in this study (for patient and tumor characteristics see Table I). The specimens obtained in the operation room were fixed in formalin for 24 h, paraffin-embedded and used for later analysis. Clinical information was obtained from the patients charts. Patients ranged in age from 45 to 83 (mean age at operation 62). As controls, three samples of healthy gingiva were analyzed. This study was approved by the Institutional Review Board and performed in accordance to the actual version of the declaration of Helsinki. Informed consent was obtained. All patients were operated between March 2005 and April 2006 at the Department of Otorhinolaryngology, Head and Neck Surgery, University Medical Center of the Johannes Gutenberg University Mainz.

### Cell culture

For our experiments the cell line UM-SCC 33 derived from squamous cell carcinoma of the head and neck (HNSCC) was used (Table I) (18,19).

The cell line was maintained in DMEM/Ham's F12 (PAA), supplemented with 5% FCS (Greiner), and antibiotic solution (penicillin 100 U/ml and streptomycin 100 μg/ml, (PAA) at 37°C in 5% CO_2_. The cell line was treated with 0.2 and 2 μM 5-Aza (Vidaza^®^) (Sigma-Aldrich) for 72 h.

### DNA isolation and bisulfite modification

Genomic DNA was extracted using the DNeasy tissue kit (Qiagen) and 2 μg DNA each were subjected for bisulfite treatment (20). Bisulfite modification of genomic DNA converts unmethylated cytidine residues to uridine residues that are then converted to thymidine during subsequent PCR. We used the Epitect^®^ Bisulfite kit (Qiagen). By the use of conversion specific primers during MSP-analysis the methylation status was then analyzed.

### MSP (methylation specific PCR)

Methylation in the region near the start codon of *MGMT*, *MLH1* and *RASSF1A* was assessed using bisulfite-treated DNA. To increase the sensitivity and specificity we applied a two-step PCR approach. First, we amplified the TSG promoter regions with the primers *MGMT*-outer-F (5′-TGG TA ATT AAG GTA TAG AG-3′, upstream), *MGMT*-outer-R (5′-CCA ATC CAC AAT CAC TCA-3′, downstream), *MLH1*-outer-F (5′-TTT TAG GAG TGA AGG AGG-3′ upstream, *MLH1*-outer-R (5′-ATA AAA CCC TAT ACC TAA TC-3′, downstream), *RASSF1A*-outer-F (5′-GAG GAG GGG ATG AAG GAG G-3′, upstream) and *RASSF1A*-outer-R (5′-CTC CAA CCA AAT ACA ACC CT-3′ downstream).

The PCR conditions were 95°C for 5 min; 40 cycles at 95°C for 30 sec, 53°C for 45 sec, and 72°C for 45 sec; and a final extension at 72°C for 10 min. Ten microliters of each sample was subjected to the second round of inner PCR amplified with the followingMSP primers: for the *MGMT* TSG, MSP-F (5′-TTT CGA CGT TCG TAG GTT TTC GC-3′ upstream) and MSP-R (5′-GCA CTC TTC CGA AAA CGA AAC G-3′, downstream) and unmethylated DNA-specific primers (UMSP), UMSP-F (5′-TTT GTG TTT TGA TGT TTG TAG GTT TTT GT-3′, upstream) and UMSP-R (5′-AAC TCC ACA CTC TTC CAA AAA CAA AAC A-3′ downstream). For the *MLH1* gene MSP analysis was performed with the following primers: MSP-F (5′-ACG TAG ACG TTT TAT TAG GGT CGT-3′, upstream), MSP-R (5′-CCT CAT CGT AAC TAC CCG CG-3′, downstream), UMSP-F (5′-TTT TGA TGT AGA TGT TTT ATT AGG GTT GT-3′, upstream) and UMSP-R (5′-ACC ACC TCA TCA TAA CTA CCC ACA-3′ downstream).

For the *RASSF1A* gene inner PCR was performed with the following primers: MSP-F (5′-GGG TTT TGC GAG AGC GCG-3′, upstream), MSP-R (5′-GCT AAC AAA GCG GAA CCG-3′ downstream), UMSP-F (5′-GGT TTT GTG AGA GTG TGT TTA G-3′, upstream) and UMSP-R (5′-CAC TAA CAA ACA CAA ACC AAA C-3′ downstream).

The PCR conditions were 94°C for 15 min; 40 cycles at 94°C for 30 sec, 62°C for 30 sec, and 72°C for 30 sec; and a final extension at 72°C for 10 min. Primers for MSP and UMSP were as previously described (10,21,22). The sequences of the primers were derived from sequences AL 355531 (*MGMT*), AC 002481 (*RASSF1A*) and AB 017806 (*MLH1*). The 81 bp and 93 bp PCR products of the *MGMT* analysis as well as the 115 bp, 124 bp products of the *MLH1* inner PCR and the 169 bp products of the *RASSF1A* analysis, respectively, were separated by electrophoresis on a 2% agarose gel and stained with ethidium bromide. Distilled water was used as negative control. Bisulfite-treated lymphocyte DNA from healthy volunteers served as a positive control for unmethylated DNA. This DNA was methylated by the use of *Sss*I methyltransferase (NEB) and used after bisulfite modification as a positive control for amplification of methylated DNA.

### DNA sequencing

The order of nucleotides in the promoter region of each gene were analyzed by DNA cycle-sequencing using the BigDye™ kit (ABI, Foster City, CA, USA). Briefly, extracted DNA samples from the cell line before and after treatment with 5-Aza have been modified with sodium bisulfite treatment. Amplification was performed by the use of M13 extended outer primers of MSP-analysis. Sequences were than determined using an ABI capillary sequencer 310.

### Immunohistochemistry

Immunohistochemical analysis of tumor samples was performed using standard procedures. In brief, formalin-fixed, paraffin-embedded tissues were used. Heat-induced antigen retrieval was performed using microwave treatment (3 times for 5 min each, 600 W in 10 mM citrate buffer, pH 6.0) of all slides after dewaxing and rehydration followed by blocking of endogenous peroxidase with 3% H_2_O_2_/methanol. Pre-incubation with 10% normal serum and 2% bovine albumin/PBS for 75 min to avoid unspecific binding, was followed by the incubation with the specific primary antibodies (MGMT, 1:20 BD Pharmingen, NY; MLH1, 1:20, BD Pharmingen; RASSF1A, 1:50, Genway, CA) for 1 h at room temperature. The slides were consecutively incubated with biotinylated secondary antibody (goat-anti-mouse, 1:250, Dako A/S, Glostrup Denmark) for 45 min and then for 30 min with streptavidin-peroxidase. The visualization of the immunoreaction was performed with 3,3′-diaminobenzidine. All washing procedures were performed in PBS; dilutions of antibodies were prepared in 2% bovine albumin/PBS at room temperature. Negative controls were performed as previously described, substituting the primary antibody with PBS.

### Quantification of the expression

For evaluation of the MGMT-expression in tumor samples we measured the stained area and intensity of each section in five fields by a computer-based image analysis method, previously described in detail by us (23). In brief, stainings were quantified by the multiplication of the stained area by the staining intensity and expressed as arbitrary units (A.U.).

### Alamar blue assay-proliferation

For determination of 5-Aza mediated functional consequences we incubated the cell line UM-SCC 33 for 72 h with 0.2 or 2 μM 5-Aza. Briefly, media were changed and 10% v/v Alamar Blue reagent (Biozol) was added to each well. The Alamar Blue^®^ assay is based on a redox indicator, changing its color from blue (oxidized) to fluorescent red (reduced). After 4-h fluorescence was measured. Color changes are a measure of cellular metabolism, corresponding to the viability and proliferative activity of the cells (24). Each experiment was repeated three times.

### Statistics

A one-sided t-test was applied to assess the statistical significance. All calculations were performed using the SAS software, version 6.12 (Statistical Analysis Systems, SAS Institute Inc., Cary, NC, USA). p-values <0.05 are indicated.

## Results

**Figure 1:**
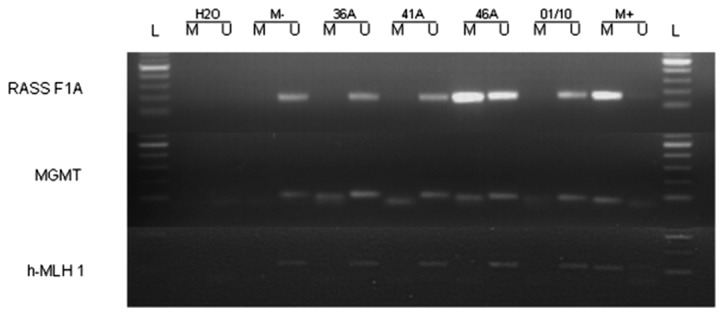
Representative results of MSP analysis of *RASSF1A*, *MGMT* and *MLH1* in biopsies obtained from primary tumors and cell lines. Bisulphite-modified DNA was amplified with primers specific for unmethylated (U) and methylated (M) DNA. Size of PCR products are 81 bp and 93 bp for *MGMT*, 115 bp and 124 bp for *MLH1* and 169 bp for *RASSF1A*. Sample numbers are indicated above the bands and correspond to those of Table I. L, 100-bp ladder.

To examine if the promoters of *MGMT*, *MLH1* and *RASSF1A* in HNSCC are methylated, we analyzed these tumor suppressor genes for their methylation status in 23 samples of primary HNSCC and one HNSCC cell line by MSP (Table II). We found that the *MGMT* promoter was methylated in 13 out of the 23 (57%) analyzed primary tumor samples and in the examined cell line. The *MLH1* promoter was found to be methylated in one out of the 23 (4%) tumor samples while the *MLH1* promoter in the UM-SCC cell line was unmethylated. MSP analysis of the *RASSF1A* promoter showed promoter methylation in 3 out of 23 (13%) tumor samples and promoter methylation in the cell line (Table II). Fig. 1 shows representative examples of the MSP-analysis. To compare the methylation status, we additionally analyzed three samples of healthy gingiva by MSP-PCR. We found that in all three samples the promoter region of *MLH1*, *RASSF1A* and *MGMT* was unmethylated. DNA sequencing verified the results of the MSP analysis in the UM-SCC 33 cell line. To analyze a possible association of hypermethylated promoter regions and decreased expression levels, we quantified the MGMT-levels by semi-quantative immunohistochemical analysis.

**Figure 2:**
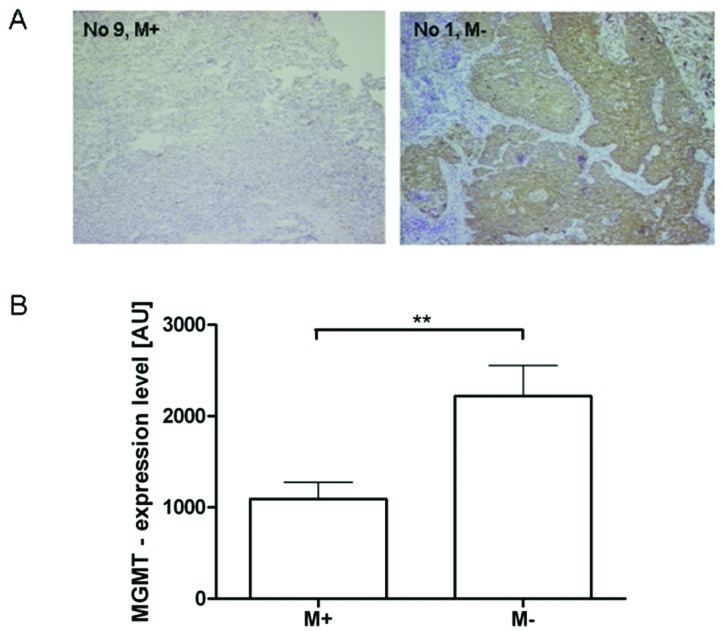
The MGMT expression in tumor samples with a hypermethylated *MGMT* promoter region is significantly lower than that in tumor samples without hypermethylation of the *MGMT* promoter region. Shown are representative immunohistochemically analyzed samples (A, ×100). Mean values ± SD in arbitrary units (A.U). Differences were calculated by t-test; ^**^p<0.01. M+, hypermethylated *MGMT* promoter region. M−, no hypermethylation of the *MGMT* promoter region.

*MGMT* expression varied from 478 to 3485 A.U. (mean 1.599±976 A.U.). M*GMT* expression statistically significantly (p<0.01) decreased in the tumor samples with a hypermethylated MGMT promoter region (Fig. 2). Due to limited tumor samples with hypermethylated *RASSF1A* and *MLH1* promoter regions, no correlation analysis of hypermethylated promoter regions and expression levels was performed.

**Figure 3:**
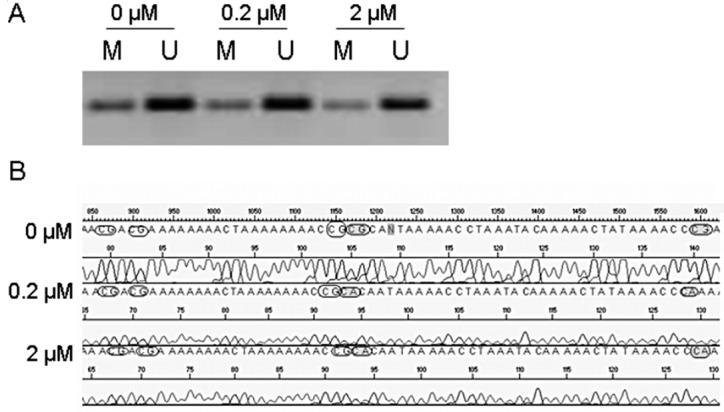
(A) MSP-analysis of UM-SCC 33 with and without 5-Aza after 72 h. No CpG-demethylation was detected. (B) DNA-sequencing of the same samples. CpGs are circled. 5-Aza treatment resulted in partial CpG-demethylation (grey-filled circles).

**Figure 4:**
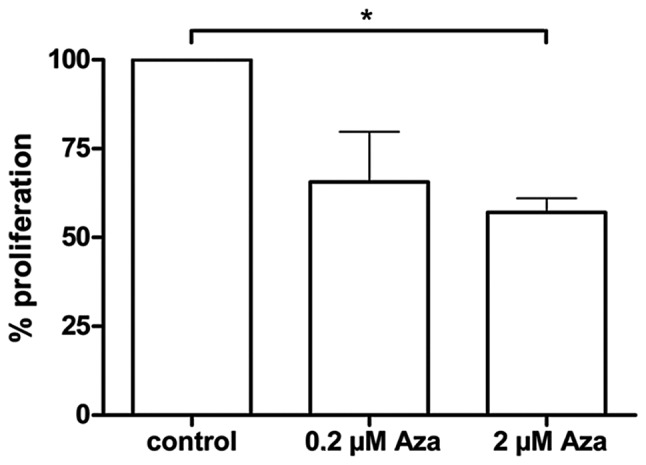
Functional impact of 5-Aza treatment on proliferation of the tumor cells. UM-SCC 33 cells were treated for 72 h with 5-Aza. The proliferative activity measured by the MTT assay 4 h after 5-Aza treatment was lowered at both 5-Aza concentrations and statistically significantly reduced at 2 μM 5-Aza. Controls were set as 100%. Shown are the mean values ± SD of three independent experiments performed in triplicate. Differences were calculated by the t-test; ^*^p<0.05.

After treatment of the UM-SCC33 cell line with 5-Aza for 72 h we observed no demethylating effect by MSP analysis (representative result is shown in Fig. 3A). However, sequence analysis showed that 5-Aza treatment led to an increased number of unmethylated CpG islands in the methylated promoter region of *RASSF1A* in the UM-SCC33 cell line (Fig. 3B). The MTT assay revealed a tendency for reduced tumor cell proliferation 4 h after treatment with 5-Aza for 72 h at a concentration of 0.2 μM (not significant) and a statistically significant reduction (p<0.05) of the proliferation at the same point of time with the 2 μM 5-Aza concentration (Fig. 4).

## Discussion

Aberrations of genomic material as well as epigenetic modifications of genome-like promoter hypermethylations may result in the deregulation of tumor suppressor genes and finally in cancer (14). In HNSCC recurrent chromosomal losses have been described (3,4,14) suggesting the involvement of several tumor suppressor genes in the genesis of HNSCC. In this study we analyzed the methylation status of three potential TSGs. *MLH1* is a mismatch repair gene, located at 3p, a chromosomal arm showing high level of allelic losses in many cases of malignancies (9,12). Several studies reported deletion or hypermethylation of the gene in hereditary colon cancer, gastric, endometrium, prostate but also HNSC cancer (21). The TSG *RASSF1A,* also located at 3p, plays a major role in the regulation of mitosis and it has been reported to be methylated in the vast majority of lung cancers and to a lesser extent in breast, ovarian and HNSC cancer (8,11,22). *MGMT* is a TSG, located at 10q, playing vital roles in preventing induction of mutations and cancer related to alkylating agents (10,13). Aberrations of *MGMT* have been reported in lung, colon, brain, liver and HNSC cancer (10).

We analyzed the methylation status of these three TSGs in tumor samples from 23 patients with HNSCC and in one cell line by MSP PCR. We found that the *MLH1* promoter was methylated in one tumor (4%) while no methylation could be observed in the cell line.

A direct association between hypermethylation of *MLH1* and cancer has been reported in colon cancer but reports on the methylation status of the same gene in HNSCC are inconclusive and have ranged from 0 to 88%. High percentage (88%) of *MLH1* methylation was reported by Liu *et al* but only in samples previous found to have loss of expression of the gene product (25). Steinmann *et al* reported that 69% of the 54 HNSCC samples showed hypermethylation of the *MLH1* promoter (26), while two studies with 96 and 57 samples did not find methylation at all (27,28). Thus, our results concerning the *MLH1* promoter methylation are in agreement with other reports. The widely divergent findings reported in the literature may be attributed to different sensitivities of the techniques as well as to insufficient DNA quality because of tissue preservation or bisulfite treatment. Furthermore, Wright and Stewart argued the importance of histological grading for *MLH1* expression, reporting that a high sample number (70%) lacked *MLH1* expression in poorly differentiated colon adenocarcinoma. Thus, the reported *MLH1* methylation status may also depend on the variety of the histological grading of a study group in HNSCC (29). Yamamoto *et al* reported a high risk of developing secondary carcinoma in the gastrointestinal tract, in patients with defective protein expression of *MLH1* (30), while in a further study a significant correlation between the methylation of mismatch repair genes and multiple oral malignancies was found (31). Since our patient cohort consisted from patients with solitary tumors, that could be a further reason for the very rare *MLH1* methylation reported in our study. Taken together, the above data suggest that aberrant *MLH1* methylation-mediated transcriptional silencing might play a role in the development of multiple synchronous or metachronous malignancies, but it may be of minor importance for the development of solitary HNSCC lesions.

The methylation of the *RASSF1A* promoter was also rare in 3 out of 23 (13%) tumor samples while the *RASSF1A* promoter was found methylated in the UM-SCC 33 cell line. These results are in accordance to the study of Dong *et al* who reported of *RASSF1A* methylation in 15% of primary HNSCC and higher frequency in cell lines (32) and to Hogg *et al* who reported of 17% *RASSF1A* methylation in HNSCC while poorly differentiated HNSCC were more commonly methylated for R*ASSF1A* than moderately and well differentiated HNSCC (8). Steinmann *et al* also report of 18% *RASSF1A* methylation in an analysis of 54 HNSCC tumor samples (26). Notably, Lo *et al* have reported 14 of 21 primary nasopharyngeal carcinomas to show *RASSF1A* promoter methylation (33). This difference in the prevalence of the *RASSF1A* methylation may reflect the known differences in the disease. Hence epigenetic inactivation of *RASSF1A* plays an important role in the development of cancer but is apparently less important in HNSCC where genes other than *RASSF1A* may be of greater importance.

The *MGMT* TSG was found in 13 out of the 23 (57%) analyzed primary tumor samples as well as in the examined cell line methylated. Accordingly, we found statistically significantly decreased protein levels of *MGMT* in the hypermethylated tumors. In the literature the incidence of *MGMT* promoter hypermethylation in HNSCC ranged from 18% to 54% (26,34,35). Frequent *MGMT* methylation can increase the sensitivity towards the mutagenic effects of DNA alkylating agents like cigarette smoke nitrosamines. On the other hand, it can facilitate the cytotoxic effects of DNA alkylating chemotherapeutics in patients with malignant astrocytomas, glioma and diffuse large B-cell lymphoma (36,37).

In gastric cancer it has been previously reported that a correlation between *MGMT* methylation and lymph node metastasis exists (38). It can be hypothesized that in HNSCC the epigenetic loss of *MGMT* function may increase the mutation rates as a result of an impaired repair of DNA damage induced by cigarette smoke nitrosamines. This may facilitate the cell to acquire an enhanced migration potential and invasiveness. In our patient cohort the *MGMT* hypermethylated samples were equally divided between nodal positive and nodal negative patients.

The frequent *MGMT* promoter hypermethylation in our study is in line with the results previously described. Our data suggest that although *MGMT* silencing by promoter hypermethylation might not be important for the development of nodal metastasis in HNSCC, it might play a role in the tumorigenesis of HNSCC, by increasing the sensitivity towards the mutagenic effects of DNA alkylating agents.

After treatment of the cell line with 5-Aza, no demethylating effect could be shown by MSP. DNA-sequencing of the promoter region of the three TSG in the UM-SCC 33 cell line, after treatment with 5-Aza showed an increase in the number of unmethylated CpG islands in the methylated promoter region of the *RASSF1A* and *MGMT* TSG in the UM-SCC 33 cell line. This can be explained through the higher sensitivity of DNA-sequencing to reveal such minor changes. The ineffective demethylation results are supported by other studies (39,40). Costello *et al* reported that only around 40% of the aberrantly methylated genes can be reactivated by 5-Aza in cultured cancer cell lines (41). Karpf and Jones (42) reported a similar result in colon cell lines, and they suggested that 5-Aza-treated tumor cell lines may no longer express activating factors required for the transcription of these particular CpG-island carrying genes.

This partial promoter demethylation was accompanied in our study by a significant decrease of proliferative activity. The observed demethylating effect of 5-Aza on the proliferative activity of UM-SCC 33 tumor cell line cannot be attributed only to demethylation of *MGMT* and *RASSF1A* promoter, since further hypermethylated TSGs might also be involved. Nevertheless, 5-Aza treatment reduces the proliferative activity of tumor cells with hypermethylated TSG promoter *in vitro* and shows that 5-Aza treatment may be feasible in the therapy of HNSCC patients with TSG promoter hypermethylation.

The value of demethylating treatment with 5-Aza has previously been shown for hematologic disorders (16,17). In these diseases the reactivation of the TSG p15, an inhibitor of cyclin-dependent kinases, is assumed to be the underlying mode of action. Even though our data revealed only limited demethylating effects through 5-Aza demethylating treatment in HNSCC patients with hypermethylation of TSGs, further studies are warranted to elucidate the potential use of demethylating treatment concepts in the treatment of HNSCC.

## Figures and Tables

**Table I:** Patient, specimen and cell line characteristics.

	Age	Gender	Site	TNM
Patient no.				
1	54	Male	Larynx	T3N0M0
2	68	Male	Oropharynx	T1N0M0
3	52	Female	Hypopharynx	T2N2bM0
4	70	Male	Floor of mouth	T1N0M0
5	61	Male	Oropharynx	T2N0M0
6	65	Male	Larynx	T3N0M0
7	65	Male	Hypopharynx	T2N2bM0
8	58	Female	Hypopharynx	T2N1M0
9	74	Male	Oropharynx	T2N2cM0
10	77	Male	Nasal sinus	T2N0M0
11	46	Male	Oropharynx	T2N1M0
12	45	Male	Oropharynx	T3N0M0
13	63	Male	Hypopharynx	T2N0M0
14	53	Male	Floor of mouth	T3N0M0
15	51	Male	Oropharynx	T2N2bM0
16	64	Male	Oropharynx	T4N2bM0
17	67	Male	Tongue	T4N0M0
18	65	Male	Larynx	T3N0M0
19	61	Male	Oropharynx	T1N1M0
20	61	Male	Larynx	T1N0M0
21	67	Female	Larynx	T1N0M0
22	61	Male	Hypopharynx	T3N2bM0
23	83	Female	Oropharynx	T2N1M0
Control no.				
24		NA	Healthy gingiva	-
25		NA	Healthy gingiva	-
26		NA	Healthy gingiva	-
Cell line				
UM-SCC 33		Female	Nasal sinus	T4N3aM0

NA, not available.

**Table II:** Analysis of *RASSF1A*, *MLH1* and *MGMT* promoter methylation by MSP in primary HNSCC and cell lines and *MGMT* expression.

	*RASSF1A* methylation	*MLH1* methylation	*MGMT* methylation	MGMT expression (A.U)
Patient no.				
1	−	−	−	3286
2	−	−	−	2596
3	−	−	−	2788
4	+	−	−	NA
5	−	−	−	1238
6	−	−	−	3485
7	−	−	+	2550
8	−	−	+	1318
9	−	−	+	532
10	−	−	−	2818
11	−	−	+	884
12	−	−	+	1491
13	−	−	+	709
14	−	−	+	865
15	−	−	−	1625
16	−	−	+	478
17	−	−	−	565
18	+	−	+	NA
19	−	+	+	952
20	+	−	−	1576
21	−	−	+	617
22	−	−	+	NA
23	−	−	+	1604
Control no.				
24	−	−	−	
25	−	−	−	
26	−	−	−	
Cell line				
UM-SCC 33	−	−	−	

NA, not available; +, yes; −, no. A.U., arbitrary units.
